# Diagnosis of Coronary Stent Dislodgement With Transthoracic Echocardiogram: A Case Report

**DOI:** 10.7759/cureus.49349

**Published:** 2023-11-24

**Authors:** Afif Hossain, Delia Cotiga, Renjit V Thomas, Maryam Afshar, Wojciech Rudzinski

**Affiliations:** 1 Cardiology, Rutgers New Jersey Medical School, Newark, USA; 2 Cardiology, VA New Jersey Healthcare System, East Orange, USA

**Keywords:** percutaneous intervention (pci), transthoracic echocardiography (tte), coronary stent dislodgment, coronary stent migration, right coronary artery (rca)

## Abstract

Coronary stent dislodgment is a rare complication of percutaneous coronary intervention (PCI). Although stent dislodgment typically occurs immediately in the intraoperative or perioperative period, it can infrequently occur subacutely in the post-operative period. Diagnosis of stent dislodgment can be seen with various cardiac imaging modalities, from transthoracic and transesophageal echocardiogram to cardiac computed tomography or magnetic resonance imaging to direct visualization on fluoroscopy during cardiac catheterization. Given the rarity of this entity, there is a lack of established common practice, gold standard for treatment, and/or procedural data. Instances are managed on a case-by-case basis, using the imaging modalities readily available at the institution and treatment modalities the interventionalist or surgeon is most comfortable with. Therefore, management of stent dislodgment consists of conservative, percutaneous, or surgical interventions on a case-by-case basis. We present a case of right coronary artery stent migration that was incidentally diagnosed with routine transthoracic echocardiogram.

## Introduction

Coronary stents have gone through several iterations over the past few decades, with significant advancement made over this time [1] and generally proven safety [2]. Despite newer technologies, stent optimization is hindered by limitations in existing stents, patient factors, and anatomical and clinical characteristics. As a result, coronary stent dislodgment can occur as a rare complication of percutaneous coronary interventions (PCIs) that may pose serious risk to the patient [3]. These cases are usually diagnosed during or soon after the procedure. We present a case of RCA stent dislodgement that was diagnosed after routine follow-up transthoracic echocardiogram (TTE) several months after the PCI.

## Case presentation

**Figure 1:**
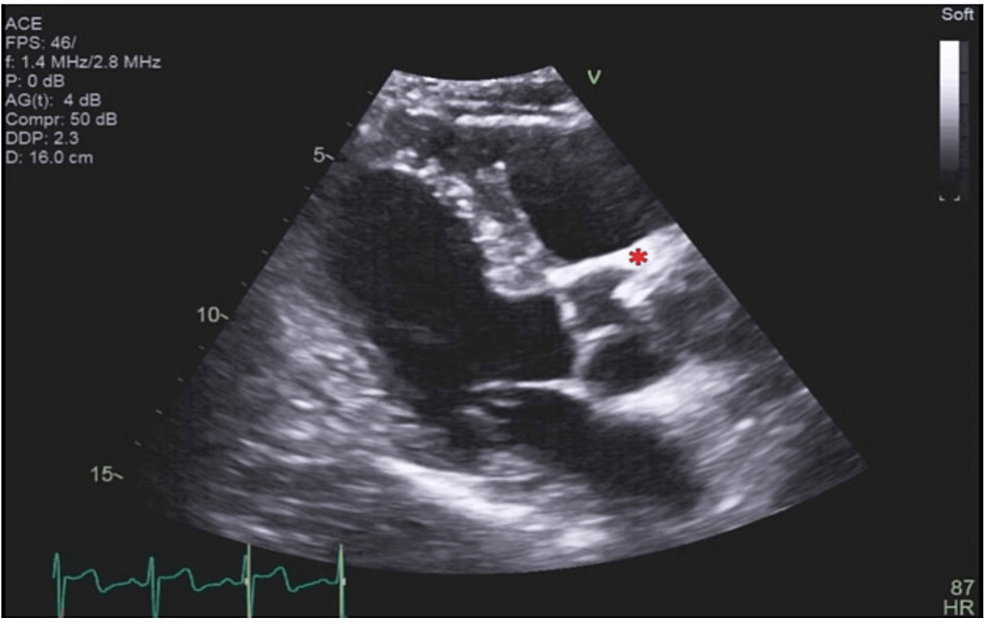
Transthoracic echocardiogram in a parasternal long-axis view Transthoracic echocardiogram (TTE) in a parasternal long-axis (PLAX) view. A long, tubular, echodense structure can be seen arising from the right coronary sinus and into the aortic root (marked with red asterisk).

**Figure 2:**
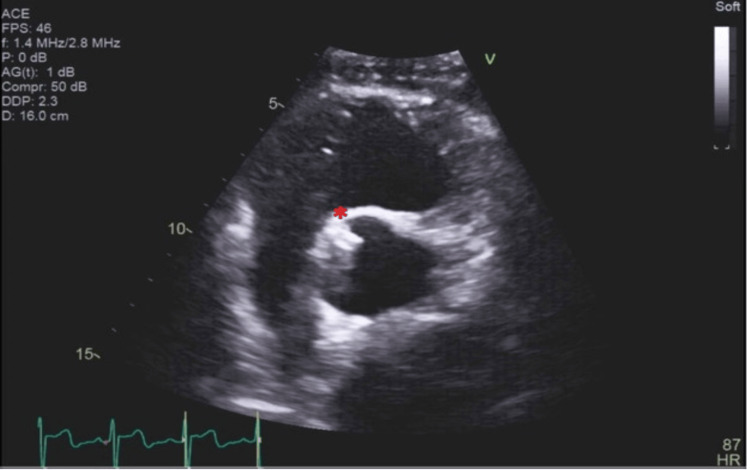
Transthoracic echocardiogram in a parasternal short-axis view Transthoracic echocardiogram (TTE) in parasternal short-axis (PSAX) view. Again, a tubular, echodense structure can be seen arising from the right coronary sinus, which protrudes into the aortic lumen at approximately the 11 o’clock position (marked with a red asterisk).

**Figure 3:**
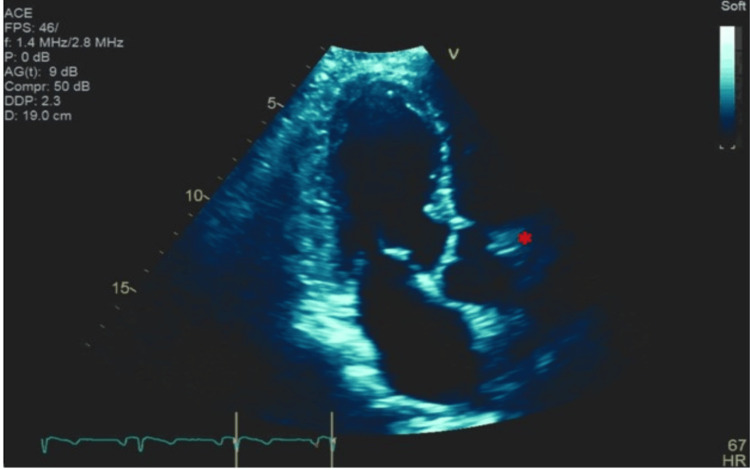
Transthoracic echocardiogram in a three-chamber view Transthoracic echocardiogram (TTE) in three-chamber view. At the level of the right coronary sinus, a tubular, echodense structure can be seen arising from the right coronary sinus, which protrudes into the aortic lumen at approximately the 1 o’clock position (marked with red asterisk).

A 58-year-old male with a history of coronary artery disease and PCI at an outside hospital reported for routine follow-up echocardiogram. The patient felt well and had no cardiovascular symptoms. Routine TTE showed a large, elongated, immobile echodensity protruding from the right coronary sinus into the aortic root, which measured approximately eight to nine millimeters in length. Given a clear lumen seen within the echodensity, we suspected dislodged right coronary artery (RCA) stent (see Figures 1-3 and Videos 1-3).

**Video 1:** Transthoracic echocardiogram in a parasternal long-axis view with stent in the longitudinal axis

**Video 2:** Transthoracic echocardiogram in a parasternal long-axis view with stent in the transverse axis

**Video 3:** Transthoracic echocardiogram in a parasternal short-axis view

**Video 4:** Transthoracic echocardiogram in a three-chamber view

**Figure 4:**
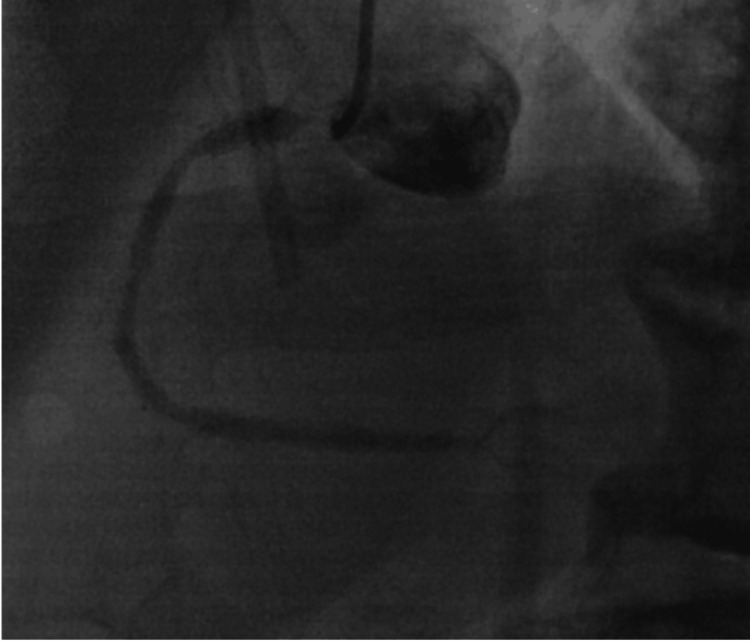
Pre-intervention coronary angiogram of the right coronary artery Coronary angiogram showing ostial right coronary artery (RCA) severe in-stent restenosis of a previously placed ostial RCA stent. There is also moderate proximal and mid RCA and distal branch vessel disease.

**Figure 5:**
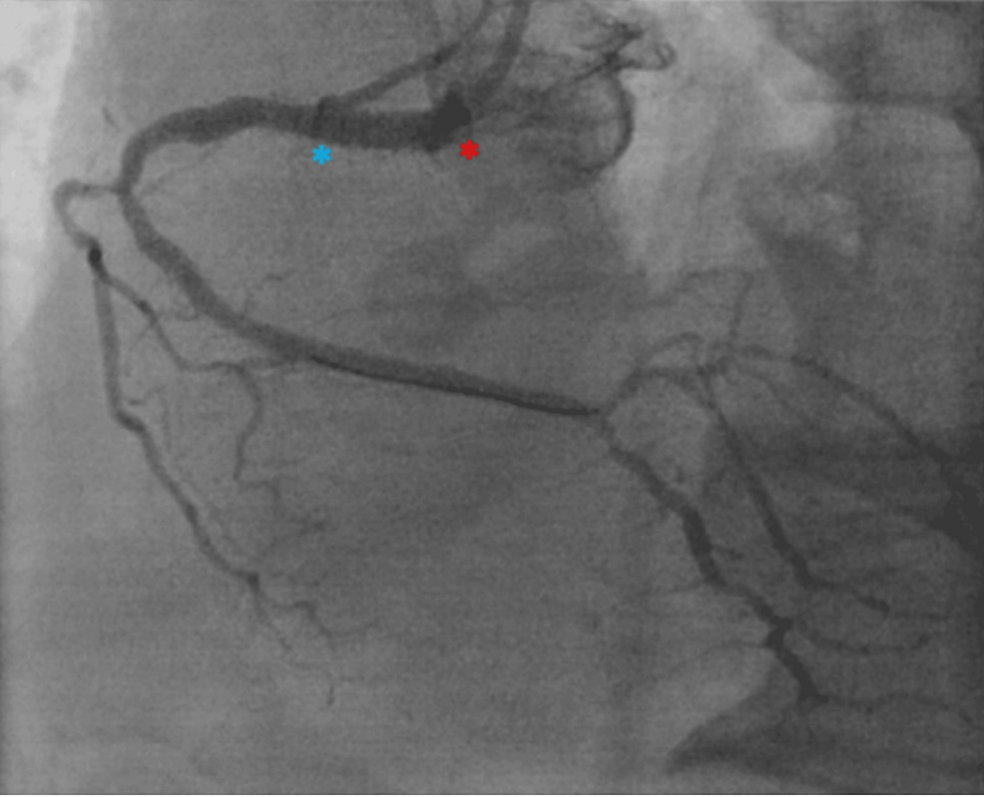
Post-intervention coronary angiogram Coronary angiogram showing right coronary artery (RCA) after percutaneous coronary intervention (PCI) of ostial RCA in-stent restenosis (ISR) with four millimeter by fifteen millimeter Xience™ Sierra drug-eluting stent (proximal end of stent marked with a red asterisk and distal end of stent marked with a blue asterisk). The stent was post-dilated with a non-compliant balloon for optimal stent expansion and apposition. Post optimization, luminal gain in ostial lesion is achieved with branch and distal vessels having greater TIMI flow than prior to intervention.

Four months before presentation, the patient underwent trans-radial coronary angiography that revealed 99% in-stent re-stenosis (ISR) of a previously placed ostial-to-proximal RCA stent (Figure 4). He underwent PCI with a 4.0 millimeter by 15 millimeter Xience™ Sierra drug-eluting stent, which was deployed to 20 atmospheres (ATM) of pressure. The lesion was post-dilated with a 4.5 millimeter non-compliant (NC) balloon to 20 ATM (Figure 5).

**Figure 6:**
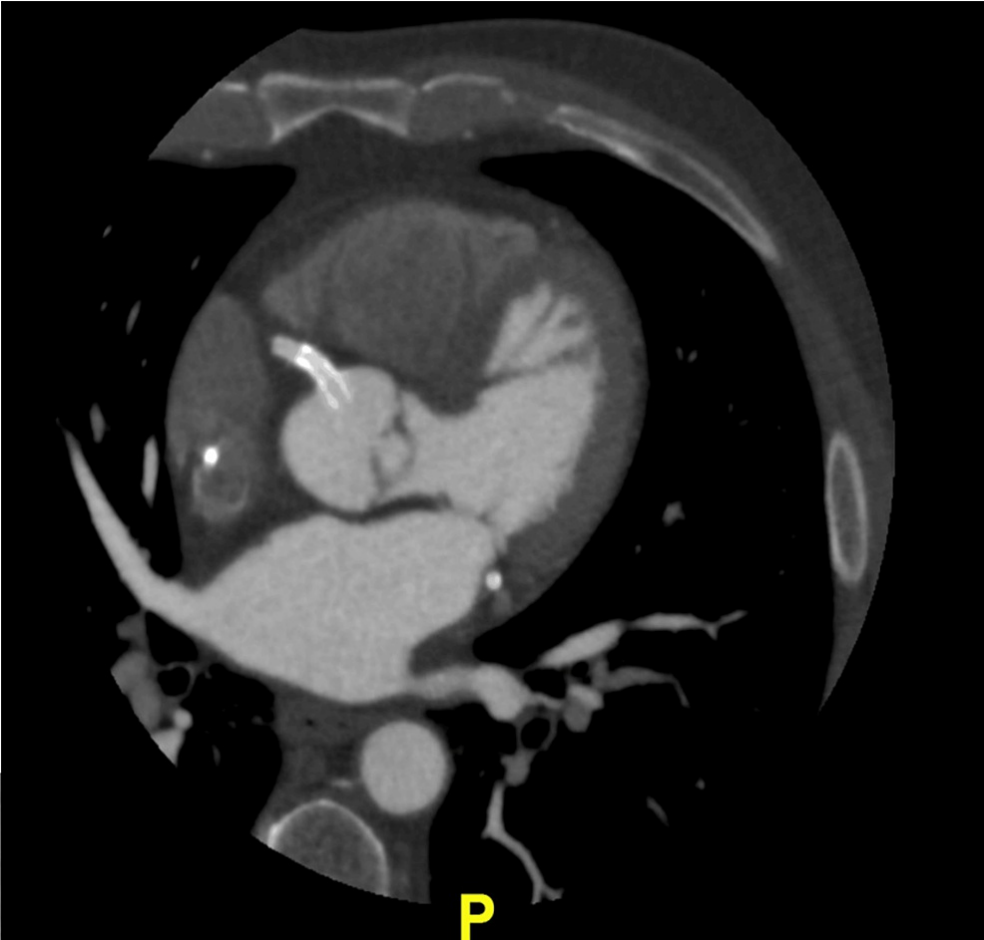
Coronary computed tomographic angiography of coronary arteries in the axial view Coronary computed tomographic angiography (CCTA) of coronary arteries in the axial view showing an echodense, tubular structure protruding from right coronary artery (RCA) into right coronary sinus and then into the aortic lumen. Based on this imaging, the stent otherwise looks patent and well-opposed to the RCA wall.

**Figure 7:**
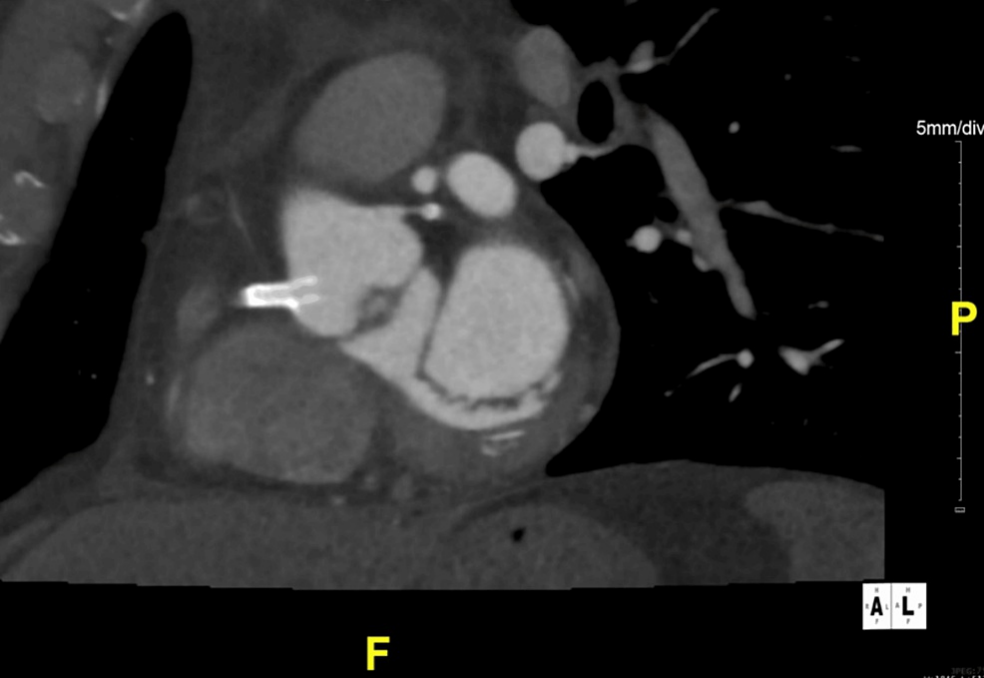
Coronary computed tomographic angiography of coronary arteries in the saggital view Coronary computed tomographic angiography (CCTA) of coronary arteries in the sagittal view showing an echodense, tubular structure protruding from right coronary artery (RCA) into right coronary sinus and then into the aortic lumen representing likely a patent stent.

Given these findings, the patient was referred for cardiac computed tomographic angiography (CCTA), which confirmed our suspicion of RCA stent dislodgment. Figures 6 and 7 represent multiplanar, reformatted CCTA images in the off-axial (Figure 6) and off-sagittal (Figure 7) planes showing a coronary stent in the ostium of RCA extending into the aortic lumen approximately eight millimeters in length. In addition, the stent appeared patent, and there was no extra-luminal thrombus associated with the stent.

After consultation with Interventional Cardiology, we treated the patient conservatively with long-term dual-antiplatelet therapy and planned to perform periodic follow-up imaging.

## Discussion

Coronary stent migration and dislodgement are rare complications of PCI with a reported incidence of 0.32-3.4% [4]. The incidence of stent migration has decreased recently due to pre-mounted stents, technical advancements, and newer procedural techniques. Several factors, including arterial tortuosity, calcification, manually crimped stents, direct stenting, complex lesions requiring crossing and recrossing, and excessive force with stent deployment, can increase the risk for stent migration [5]. For example, direct stenting can lead to stent migration due to increased resistant to stent advancement through a target lesion, when compared to pre-dilation. Classically, manually crimped stents compared to pre-mounted stents had higher risk for stent migration [5]. Procedural components, like non-coaxial engagement or deploying a deformed stent, may also increase the risk for stent migration [5].

Stent migration can cause severe, life-threatening complications, such as myocardial infarction, coronary thrombosis, stent embolization, and/or cerebroembolism [6]. Although coronary stent migration is usually diagnosed at the time of coronary intervention, delayed migrations can occur and can be successfully detected with echocardiogram. In our case, routine TTE led to further diagnostic imaging that later confirmed coronary stent migration. When stent migration occurs, treatments can be minimally invasive with interventional techniques, such as snaring, ballooning, or crushing, surgical for bypass or surgical removal, and lastly, for lower risk cases, conservative management with anti-platelet therapy, medication to optimize stent epithelialization, and serial follow-up with imaging [7, 8]. Since each method has its merits and consequences, the treatment needs to be performed on a case-by-case basis to optimize patient outcomes.

## Conclusions

Stent dislodgement and embolization are uncommon complications of PCI that can lead to serious intra- and post-PCI complications. Post-PCI, stent dislodgment can occur acutely or subacutely. Despite the best stent optimization, stent migration can infrequently occur. Advanced imaging techniques, such as coronary computed tomographic angiography, are imaging modalities of choice to diagnose stent dislodgment. However, well-performed transthoracic echocardiogram can successfully detect ostial coronary stent dislodgement as well.
